# Supplementary material for: Archean phosphorus liberation induced by iron redox geochemistry
Source: Nat Commun. 2018 Apr 9;9:1346. doi: 10.1038/s41467-018-03835-3 (PMC5890264; doi:10.1038/s41467-018-03835-3)
Supplement: Supplementary file 1 — Supplementary Information [file 41467_2018_3835_MOESM1_ESM.pdf]

Archean phosphorus liberation induced by iron redox geochemistry

Supplementary Information

Herschy et al.

Supplementary Table 1. Measured phosphite salt solubility characteristics at 298 K including solubility product (K_{SP}) and estimated number of moles from addition of excess phosphite salt to water. Data acquired from ICP-OES, in solutions with pH 7. For contrast the equivalent predicted solubility of similar phosphate minerals at pH 7 is also given.

Mineral/Salt		K_{SP}	Molarity of P
$CaHPO_3$		4.3×10^{-6}	0.002
$MgHPO_3$		5.5×10^{-5}	0.007
$Fe_2(HPO_3)_3$		5.0×10^{-21}	0.0001
$FeHPO_3$		1.3×10^{-8}	0.0001
$Al(H_2PO_3)(HPO_3)$		2.1×10^{-9}	0.002
Apatite	$Ca_5(PO_4)_3OH$		5×10^{-5}
Brushite[1]	$CaHPO_4{\bullet}2H_2O$		0.0008
Newberyite[2]	$MgHPO_4{\bullet}3H_2O$		0.0017
Vivianite[3]	$Fe_3(PO_4)_2{\bullet}8H_2O$		4×10^{-6}
Strengite[4]	$FePO_4{\bullet}2H_2O$		3×10^{-11}
Variscite[5]	$AlPO_4{\bullet}2H_2O$		2×10^{-5}

[1]Ferreira et al. 2003[SI.26], [2]Babić-Ivančić et al. 2006 [SI.27], [3]Al-Borno and Tomson 1994 [SI.25], Nriagu 1972 [SI.28], [5]Taylor and Gurney 1964 [SI.29]

Supplementary Table 2. Experimental fluids and starting conditions.

Sample ID	HPO_3 (mM)	Fe (mg)	Total volume (mL)	pH	Atmosphere	Temperature (°C)
This study						
A2	30	572	20	7	Air	22 ± 2
A5	30	572	20	7	Air	4 ± 2
A9	30	-	20	7	Air	4 ± 2
A10	30	-	20	7	Air	22 ± 2
A12	-	572	20	7	Air	22 ± 2

Supplementary Table 3. Abundances of phosphate produced by the oxidation of phosphite by iron powder.

Sample ID	Time (Days)	Dissolved PO_4 (μmole)	PO_4 associated with IOH (μmole)	Total PO_4 (μmole)	HPO_3 oxidation (%)	Formation of red IOH
A2	0	0	0	0	0.0	
	3	3	10	14	2.3[a]	+
	35	4	9	13	2.2[a]	++++
	1,901	9	36	46	7.6[a]	+++++
A5	0	0	0	0	0.0	
	3	7	0	7	1.2	
	35	8	0	9	1.4	
	1,901	600	1	601	100.2	+
A9	0	0	-	0	0.0	
	3	0	-	0	0.0	
	35	0	-	0	0.0	
	1,901	1	-	0	0.1	
A10	0	0	-	0	0.0	
	3	0	-	0	0.0	
	35	0	-	0	0.0	
	1,901	2	-	2	0.3	
A12	0	0	0	0	0.0	
	3	0	0	0	0.0	
	35	0	0	0	0.0	
	1,901	0	0	0	0.0	

[a] The percentage of the oxidation of HPO_3^{2-} of A2 sample is 600 micromoles less phosphate formed.

Supplementary Fig. 1.

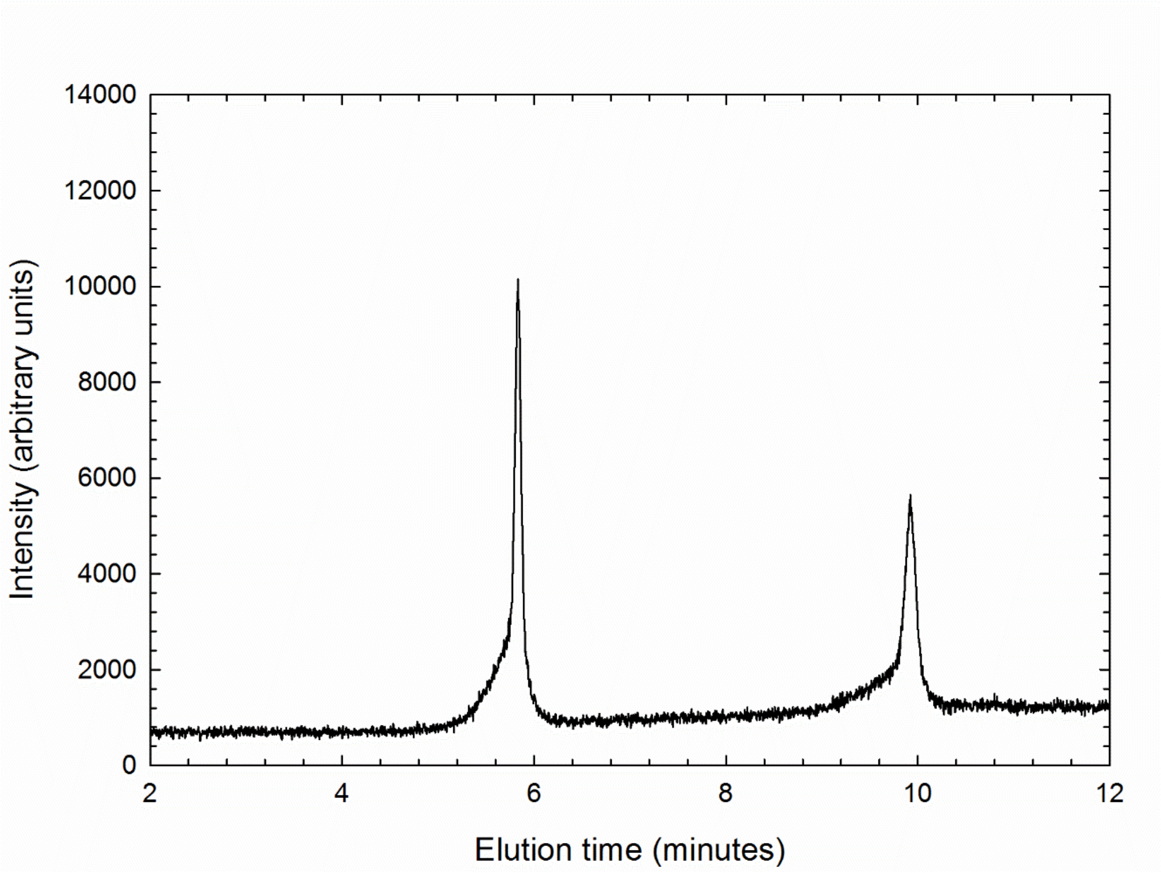

Chromatogram of phosphorus standards phosphite (peak at 6 minutes) and phosphate (peak at 10 minutes), both at 10^{-6} M.

Supplementary Fig. 2.

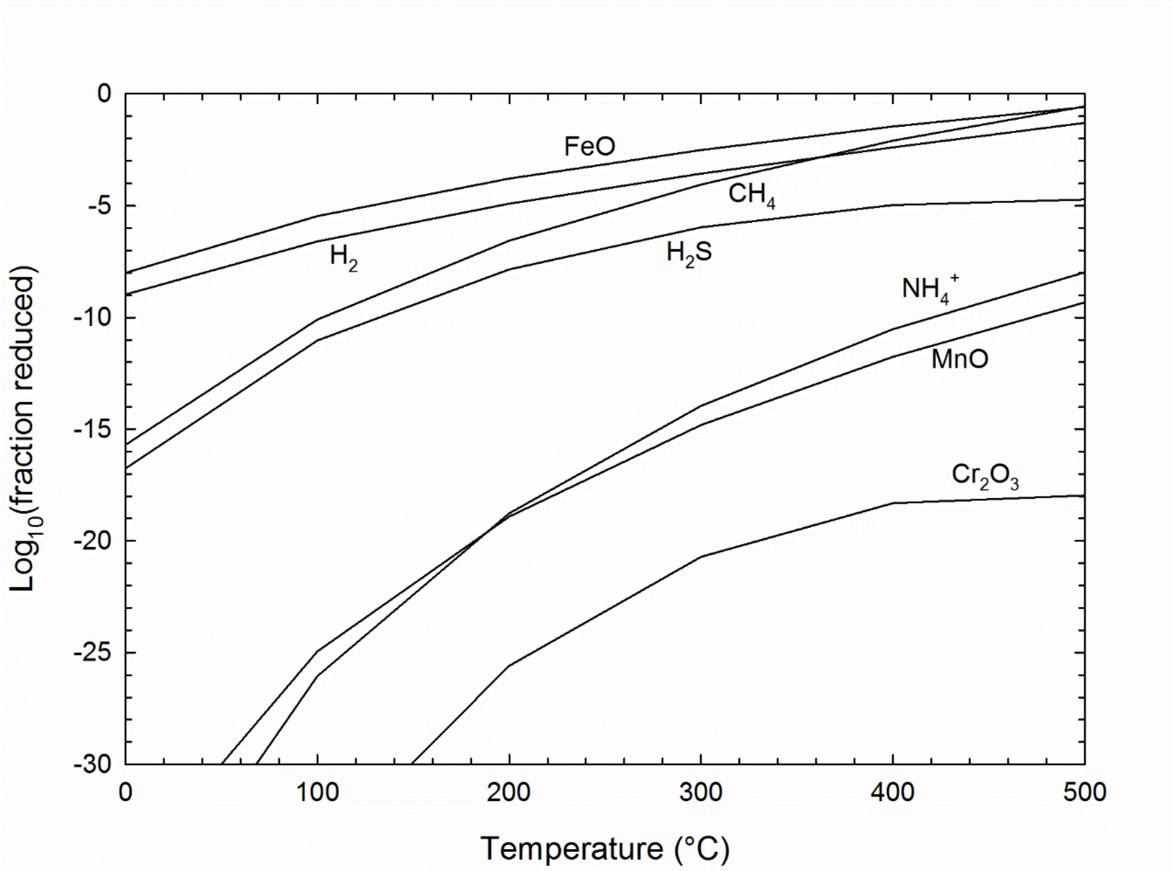

Thermodynamic models of the fraction of P reduced using various reducing agents as a function of temperature.

Supplementary Fig. 3.

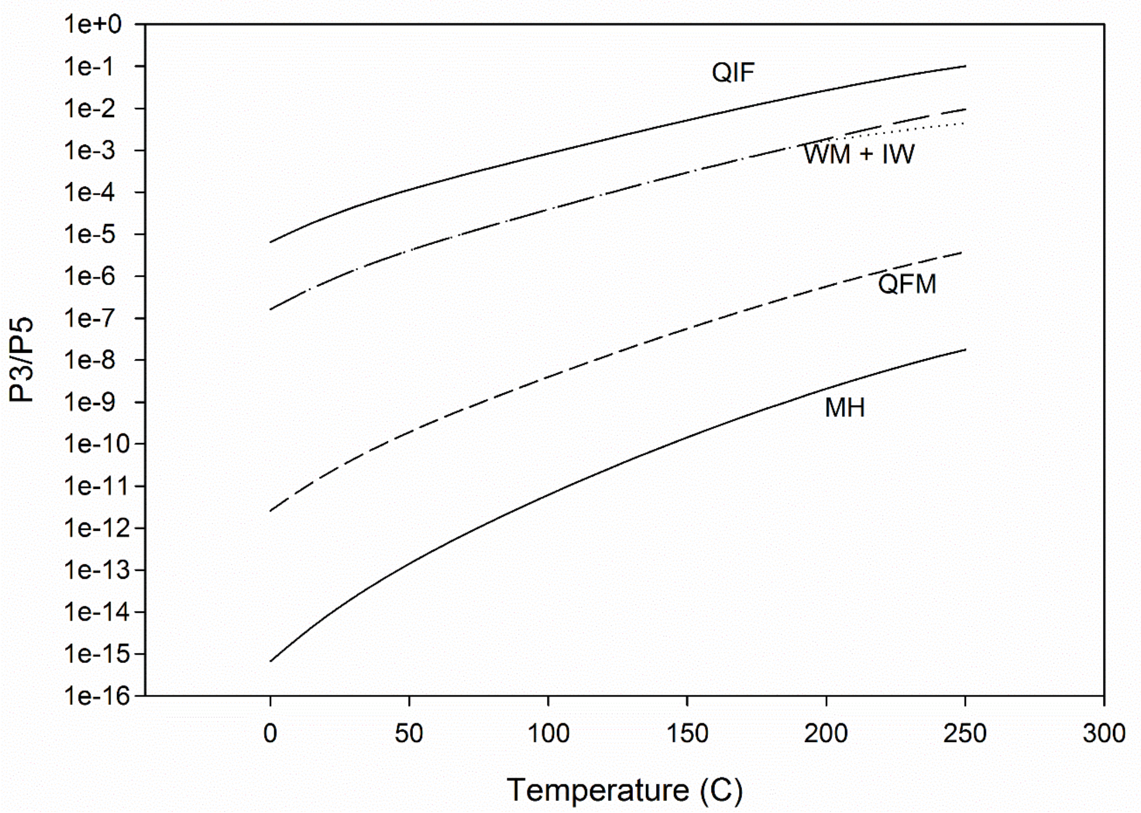

Fraction of P reduced (P3) relative to phosphate (P5) vs. temperature, depending on mineral redox buffer system present. QIF is quartz-iron-fayalite, WM is wüstite-magnetite, IW is iron-wüstite, QFM is quartz-fayalite-magnetite, and MH is magnetite-hematite.

Supplementary Fig. 4.

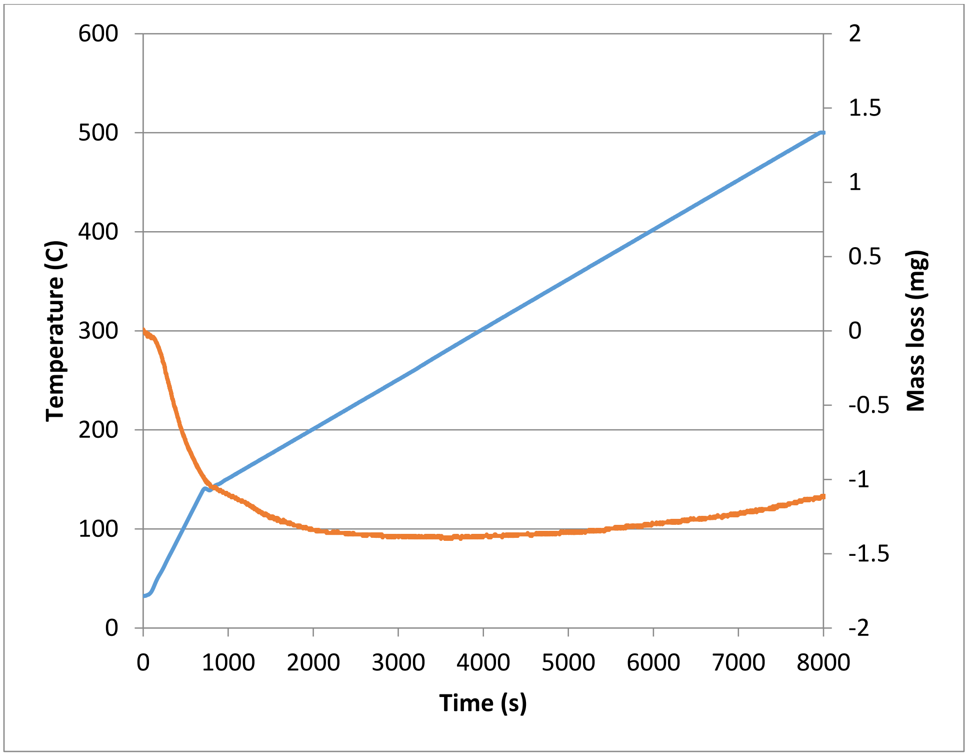

Thermogravimetric analysis of the oxidation of $Fe(H_2PO_3)_2{\cdot}2H_2O$ under air. The initial mass was 9.700 mg. The loss of mass until about 270°C corresponds to dehydration of the two waters of crystallization, followed by a slow partial oxidation of phosphite (about 25% by 500°C, the experiment's completion temperature). The activation energy of the oxidation of $Fe(H_2PO_3)_2$ is calculated using the Arrhenius relationship as 42 kJ/mol, with a frequency of 0.071 s^{-1}. At 298 K, the oxidative half-life of this compound is estimated as ~6 years under air. The reaction is presumed to be first order with respect to O_2 partial pressure. At lower partial pressure of O_2 (e.g., [6]), the rate should decrease proportional to P_{O2}. Prior to the rise of oxygen ($<10^{-5}\times$ present atmospheric level), the oxidation half-life would have been 600,000 years.

Supplementary Fig. 5.

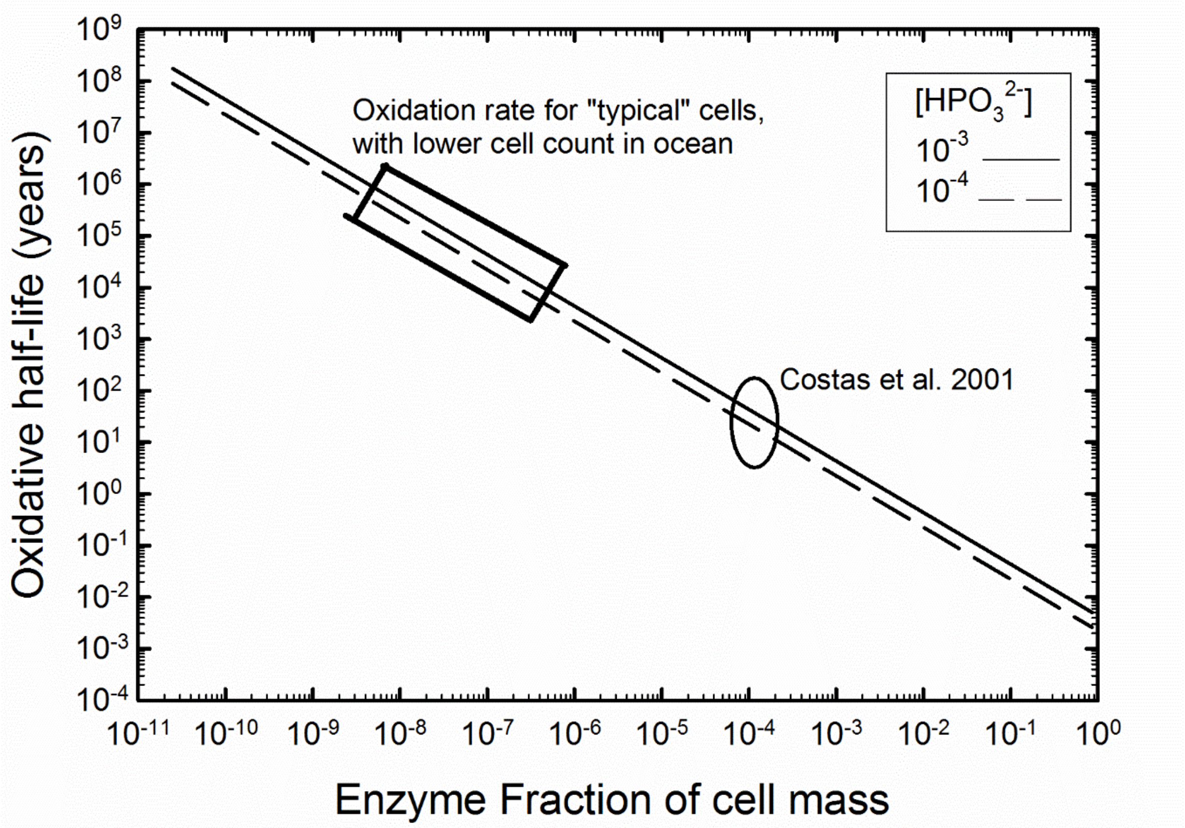

Estimates of the oxidation half-life of a solution of phosphite as a function of fraction of ptxD enzyme present in microorganisms capable of oxidizing phosphite. Two initial concentrations of phosphite are shown: 10^{-3} and 10^{-4} M. Costas et al. (2001) retrieved[7] about 2 mg of ptxD from 20 g of cell mass, after overexpression of the enzyme in *E. coli*, and this should represent the maximum enzyme quantity under typical ecological conditions. Likely, the biological oxidation rate should decrease by a factor of 100-10^4× because of a decreased cell count (about 10^4 cells/mL vs. 10^6 cells/mL through the depth of the ocean, see [8]) and because microbes should not have such a high concentration of the ptxD enzyme (a factor of 10-100× less).

Supplementary Fig. 6.

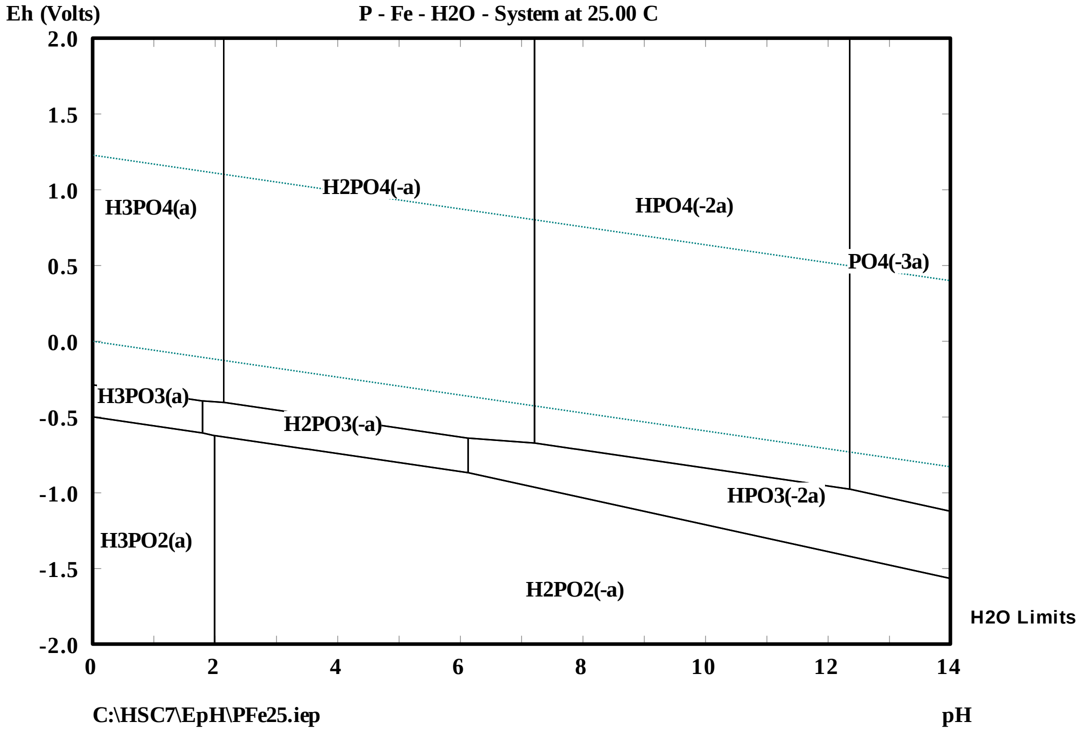

Eh-pH diagrams for P aqueous speciation. Total P and Fe were 10^{-8} M. Allowed species include Fe, FeO, Fe_2O_3, Fe_3O_4, $FePO_4$, $FePO_4 \bullet 2H_2O$, Fe^{3+}, Fe^{2+}, $FeHPO_4^{+}$, $Fe_3(PO_4)_2$(aq), H_3PO_4(aq), H_3PO_3(aq), H_3PO_2(aq), $H_2PO_4^{-}$, $H_2PO_3^{-}$, $H_2PO_2^{-}$, HPO_4^{2-}, HPO_3^{2-}, and PO_4^{3-}. The dashed lines are the oxidizing conditions of present day atmosphere (top), and most reducing conditions generally found in contact with water (bottom). Under no conditions are phosphite or hypophosphite considered thermodynamically stable in water at room temperature.

Supplementary Fig. 7

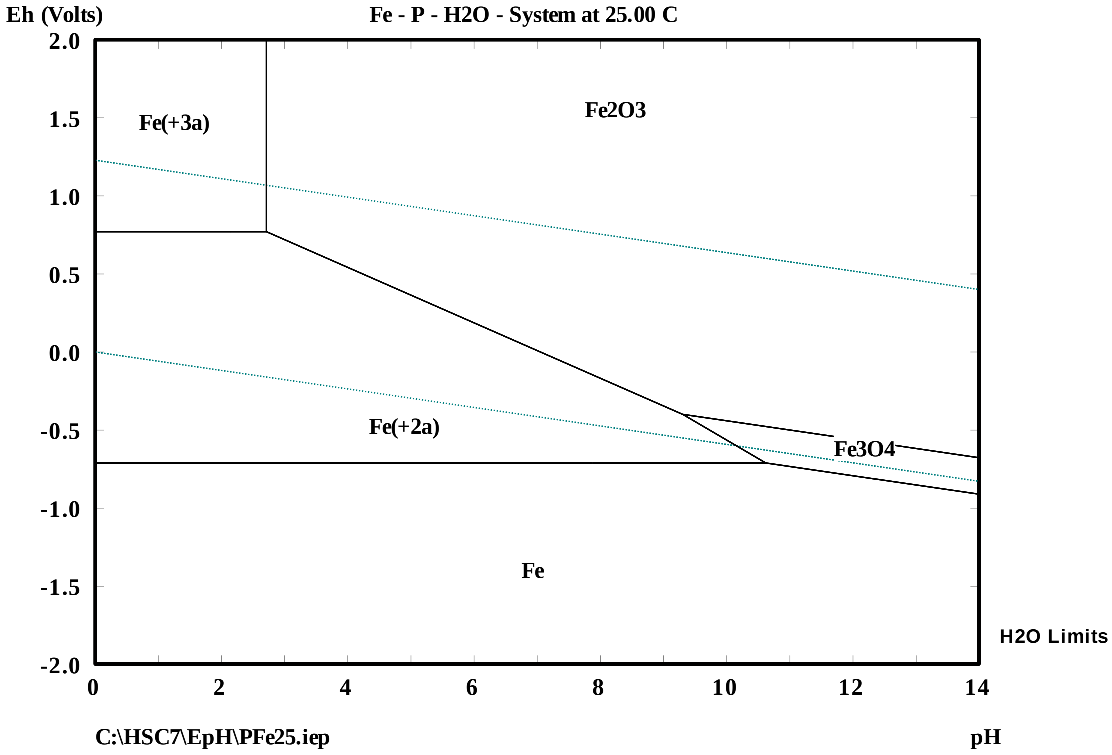

Eh-pH diagrams for Fe aqueous speciation. Details as per Supplementary Fig. 6.

Supplementary References

1. Ferreira, A., Oliveira, C. and Rocha, F., The different phases in the precipitation of dicalcium phosphate dihydrate. *Journal of Crystal Growth*, *252*(4), pp.599-611 (2003).
2. Babić-Ivančić, V., Kontrec, V.J., Brečević, L. and Kralj, D., Kinetics of struvite to newberyite transformation in the precipitation system MgCl 2–NH 4 H 2 PO 4–NaOH–H 2 O. *Water research*, *40*(18), pp.3447-3455 (2006).
3. Al-Borno, A. and Tomson, M.B., The temperature dependence of the solubility product constant of vivianite. *Geochimica et Cosmochimica Acta*, *58*(24), pp.5373-5378 (1994).
4. Nriagu, J.O., Solubility equilibrium constant of strengite. *American Journal of Science*, *272*(5), pp.476-484 (1972).
5. Taylor, A.W. and Gurney, E.L., Solubility of Variscite. *Soil Science*, *98*(1), pp.9-13 (1964).
6. Bekker, A. and Holland, H.D., Oxygen overshoot and recovery during the early Paleoproterozoic. *Earth and Planetary Science Letters*, *317*, pp.295-304 (2012).
7. Costas, A. M. G., White, A. K., and Metcalf, W. W., Purification and characterization of a novel phosphorus-oxidizing enzyme from Pseudomonas stutzeri WM88. *Journal of Biological Chemistry*, *276*(20), pp. 17429-17436, (2001).
8. Karner, M. B., DeLong, E. F., and Karl, D. M., Archaeal dominance in the mesopelagic zone of the Pacific Ocean. *Nature*, *409*(6819), pp. 507-510, (2001).
